# Identification of sitagliptin binding proteins by affinity purification mass spectrometry: Sitagliptin binding proteins identified by affinity purification mass spectrometry

**DOI:** 10.3724/abbs.2022142

**Published:** 2022-10-09

**Authors:** Xue-Ning Wang, Byu-Ri Sim, Hong Chen, Yun-Xiao Zheng, Jun-Biao Xue, Lei Wang, Wei-Sha Kong, Kuan Zhou, Shu-Juan Guo, Jing-Li Hou, Jiong Zhang, He-Wei Jiang, Sheng-Ce Tao

**Affiliations:** 1 Shanghai Center for Systems Biomedicine Key Laboratory of Systems Biomedicine (Ministry of Education) Shanghai Jiao Tong University Shanghai 200240 China; 2 State Key Laboratory of Microbial Metabolism Joint International Research Laboratory of Metabolic and Developmental Sciences MOE-LSB & MOE-LSC School of Life Sciences and Biotechnology Shanghai Jiao Tong University Shanghai 200240 China; 3 Instrumental Analysis Center Shanghai Jiao Tong University Shanghai 200240 China; 4 Inflammation and Immune Mediated Diseases Laboratory of Anhui Province School of Pharmacy Anhui Medical University Hefei 230032 China; 5 Key Laboratory of Organofluorine Chemistry Center for Excellence in Molecular Synthesis Shanghai Institute of Organic Chemistry University of the Chinese Academy of Sciences Chinese Academy of Sciences Shanghai 200032 China.

**Keywords:** AP-MS, drug-target interaction, sitagliptin, SILAC, SPR, type 2 diabetes mellitus

## Abstract

Type 2 diabetes mellitus (T2DM) is recognized as a serious public health concern with increasing incidence. The dipeptidyl peptidase-4 (DPP-4) inhibitor sitagliptin has been used for the treatment of T2DM worldwide. Although sitagliptin has excellent therapeutic outcome, adverse effects are observed. In addition, previous studies have suggested that sitagliptin may have pleiotropic effects other than treating T2DM. These pieces of evidence point to the importance of further investigation of the molecular mechanisms of sitagliptin, starting from the identification of sitagliptin-binding proteins. In this study, by combining affinity purification mass spectrometry (AP-MS) and stable isotope labeling by amino acids in cell culture (SILAC), we discover seven high-confidence targets that can interact with sitagliptin. Surface plasmon resonance (SPR) assay confirms the binding of sitagliptin to three proteins, *i*. *e*., LYPLAL1, TCP1, and CCAR2, with binding affinities (K _D_) ranging from 50.1 μM to 1490 μM. Molecular docking followed by molecular dynamic (MD) simulation reveals hydrogen binding between sitagliptin and the catalytic triad of LYPLAL1, and also between sitagliptin and the P-loop of ATP-binding pocket of TCP1. Molecular mechanics Poisson-Boltzmann Surface Area (MMPBSA) analysis indicates that sitagliptin can stably bind to LYPLAL1 and TCP1 in active sites, which may have an impact on the functions of these proteins. SPR analysis validates the binding affinity of sitagliptin to TCP1 mutant D88A is ~10 times lower than that to the wild-type TCP1. Our findings provide insights into the sitagliptin-targets interplay and demonstrate the potential of sitagliptin in regulating gluconeogenesis and in anti-tumor drug development.

## Introduction

Drugs act by interacting with target proteins to activate or inhibit the targets’ biological processes. Drug targets mainly include enzymes, nuclear receptors, G protein-coupled receptors (GPCRs), and ion channels [ 1, 2] . Instead of the one target-one drug model, drugs usually have multiple targets, and therefore identification of drug-target interactions (DTIs) is an indispensable step in drug development [2]. The advantage of drugs with multiple targets is that a known drug can potentially bind to targets other than the initially designed one, and therefore it may be applied for treating other diseases [3]. For example, drug repositioning, which refers to identifying novel targets for existing drugs, can effectively simplify the regulatory procedures for introducing an originally approved drug on the market [3]. Thalidomide for treatment of complications of leprosy and aspirin for anti-platelet aggregation are excellent examples. However, multiple targets can lead to off-target effects, on which unexpected DTIs may cause harmful side effects.

Identification and validation of new drug-binding proteins can help us discover new therapeutic effects as well as molecular mechanisms of side effects of approved drugs. Affinity purification mass spectrometry (AP-MS) is widely used for the identification of DTIs [4]. In addition, acquiring reliable kinetic parameters for validating and characterizing DTIs is also an important part for discovering candidate drugs and targets. A variety of label-free technologies for measuring DPIs have been widely used, which include surface plasmon resonance (SPR), biolayer interferometry (BLI), isothermal titration calorimetry (ITC), and analytical ultracentrifugation (AUC) [5]. Among them, SPR has been widely used for real-time characterization of biomolecular interactions for over two decades [6].

Diabetes mellitus (DM) has posed a major global health threat, and Asia is the epicenter [7]. It is estimated that one in 11 adults are diagnosed with DM; among them over 90% have type 2 diabetes mellitus (T2DM) [8]. The driving force of hyperglycemia in T2DM is insulin resistance, *i*. *e*., impaired insulin action and decreased insulin secretion response to glucose [9]. Currently, optimizing glycemic control is recommended as a key point in the treatment of T2DM [10]. Antidiabetic drugs mainly include Metformin, dipeptidyl peptidase-4 (DPP-4) inhibitors, sodium-glucose cotransporter-2 (SGLT-2) inhibitors, α-glucosidase inhibitors, glucagon-like peptide 1 (GLP-1) receptor agonists, sulfonylurea, and thiazolidinedione [11]. Metformin is a classic, first-line therapy that has been applied in clinical practice for decades to treat insulin resistance in T2DM patients [12]. Sitagliptin, the first approved DPP-4 inhibitor considered to have fewer side effects than metformin, is firmly established as important mono- or combination therapy (with other anti-hyperglycemic drugs) in T2DM adult patients [13]. Sitagliptin targets membrane protein DPP-4, exhibiting potent and highly selective inhibition of DPP-4 inactivation of the endogenous incretin hormones GLP-1 and glucose-dependent insulinotropic polypeptide (GIP) [14]. This inhibition increases active incretin levels, leading to glucose-dependent increase in insulin release and decrease in glucagon.

Clinical trials have proved that sitagliptin is generally well tolerated with mild to moderate intensity of adverse events, however, a few studies reported that sitagliptin is likely associated with angio-oedema and worsening renal function [ 15, 16] . In addition, more and more investigations have revealed the pleiotropic effects of sitagliptin, including the potential use in anti-tumor therapy, cell autophagy regulation, fatty acid metabolism regulation, and cardiovascular protection [ 17– 20] . For example, Hollande *et al*. [17] demonstrated that administration of sitagliptin limits tumor growth by enhancing chemokine CCL11 and increasing eosinophil migration in hepatocellular carcinoma and breast cancer models. Fan *et al*. [20] found that sitagliptin suppresses the activation of p38/NF-κB signaling and protects against hypoxia/reoxygenation (H/R)-induced cardiovascular diseases. These studies suggest that sitagliptin participates in a variety of biological process, which may include interactions with other proteins. However, few studies have focused on the global identification of sitagliptin interactors, and the auxiliary functions as well as the potential side effects of sitagliptin are not fully understood.

In the present study, by performing biotin-streptavidin AP-MS combined with SILAC, we identified seven high-confidence cellular targets of sitagliptin. We further showed that three proteins (LYPLAL1, CCAR2, and TCP1) had weak binding affinities with sitagliptin. Based on the available crystal structure of LYPLAL1 and TCP1, structural analysis predicted the mechanism of sitagliptin binding to LYPLAL1 and TCP1. For CCAR2, we constructed a protein-protein interaction network which showed seven biological processes (BPs) that CCAR2 participated in. Overall, our work revealed the possibility of sitagliptin binding to cellular proteins, suggesting that sitagliptin may have the potential to regulate gluconeogenesis and to be used in tumor therapy.

## Material and Methods

### Cloning

To generate expression clones, donor vectors containing fragments of *LYPLAL1*, *NAA50*, and *RBM34* (Thermo Scientific, Waltham, USA) were shuttled into pDEST15 vectors by the LR reaction using the Gateway LR Clonase II Enzyme Mix (Thermo Scientific) according to manufacturer’s instructions. Protein sequences of CCAR2, SARS1, and TCP1 were downloaded from GenBank, and corresponding DNA fragments were codon-optimized and synthesized by Sangon Biotech (Shanghai, China). C-terminal Flag-His _6_ tag fused SARS1 and TCP1 were cloned into pET32a, while C-terminal Flag tag fused CCAR2 was cloned into pGEX-4T-1. All plasmids were transformed into *Escherichia coli* BL21 (DE3) strains to construct transformants and express proteins.

### Protein expression and purification

Recombinant *E*. *coli* BL21 was cultured in 600 mL LB medium at 37°C to an OD _600_ of 0.6. Proteins were expressed by induction with 0.4 mM isopropyl-β- *D*-thiogalactoside (IPTG) at 18°C overnight. To purify the His _6_-tagged proteins, cell pellets were resuspended and lysed by a high-pressure cell cracker (Union-biotech, Shanghai, China) in lysis buffer (50 mM Tris-HCl, pH 8.0, containing 500 mM NaCl and 10 mM imidazole). Following centrifugation at 15,000 *g* for 20 min at 4°C, the supernatant was collected and purified using Ni-NTA agarose (QIAGEN, Hilden, Germany). After wash with lysis buffer, proteins were eluted with 50 mM Tris-HCl buffer (pH 8.0), containing 500 mM NaCl and 300 mM imidazole. To purify GST-tagged proteins, cell pellets were resuspended and lysed by the high-pressure cell cracker in lysis buffer (50 mM Tris-HCl, pH 8.0, containing 500 mM NaCl and 1 mM DTT). The supernatant obtained by centrifugation were incubated with Glutathione Sepharose 4 Fast Flow (Cytiva, Waltham, USA). Then bound proteins were washed with lysis buffer and eluted with 50 mM Tris-HCl buffer (pH 8.0), containing 500 mM NaCl, 1 mM DTT and 40 mM glutathione. Purified proteins were assessed by SDS-PAGE and Coomassie brilliant blue (CBB) staining.

### SILAC labeling and lysate preparation

For the labeling of cells by SILAC, HEK293T cells purchased from the Cell Bank of the Chinese Academy of Sciences (Shanghai, China) were cultured in DMEM (deficient in L-arginine and L-lysine), supplemented either with natural L-lysine and arginine, or with L-lysine-2HCl ( ^13^C _6_) and L-arginine-HCl ( ^13^C _6_^15^N _4_) at the same concentrations. The medium was supplemented with 10% fetal bovine serum (FBS; Excell Bio, Taicang, China) and 1% penicillin-streptomycin (Invitrogen, Carlsbad, USA), and cells were cultured at 37°C in an incubator with 5% CO _2_. Cells were digested with 0.25% trypsin and were passaged and cultured for 12–16 h before transfection. Cell transfection was performed using Lipofectamine 2000 transfection reagent (Invitrogen) following the manufacturer’s instructions. After transfection, cells were incubated for 36–48 h. For the preparation of cell lysates, cells were washed three times with PBS and lysed in M-PER mammalian protein extraction reagent (Thermo Scientific). Cell extracts were incubated on ice for 40 min and centrifuged at 16,000 *g* for 10 min to pellet the cell debris. The supernatant was collected and stored at –80°C for future use.

### Affinity purification and SILAC analysis

Heavy and light stable isotope-labeled HEK293T cells were lysed and then incubated with 2 μM biotin-PEG5-sitagliptin and biotin-PEG5, respectively. The “Heavy” and “Light” lysates were then combined, and the mixture was incubated with streptavidin beads (Thermo Scientific) at 4°C overnight. After incubation, streptavidin beads were washed to remove non-specific binding proteins and stored at –80°C for quantitative liquid chromatography-mass spectrometry (LC-MS/MS) analysis.

### LC-MS/MS analysis

Proteins enriched by streptavidin beads were reduced by incubation with DTT (final concentration of 10 mM) at 37°C for 1 h and were then alkylated by incubation with iodoacetamide (final concentration of 25 mM) in the dark for 20 min. Proteins were then digested with trypsin (1:30 protein-to-enzyme ratio) at 37°C overnight, followed by rinsing with 200 μL of 50 mM NH _4_HCO _3_. All the supernatant was collected and desalted using a MonoSpin C18 care desalting column (GL Science, Tokyo, Japan) according to the manufacturer’s instructions. Then the tryptic peptide digests of the proteins were analyzed on the EASY-nL 1200 system connected to a Q Exactive plus mass spectrometer (Thermo Scientific). Raw MS spectra were processed by using Protein Discoverer 2.4 software (Thermo Scientific). SILAC 2plex (Arg10 Lys6) method was selected for quantification analysis. The following search parameters were employed: full tryptic specificity was required, and two missed cleavages were allowed; Carbamidomethylation (C) was set as fixed modification; Oxidation (M), Deamidation (NQ), and Acetylation (N-terminus) were set as variable modifications. Precursor ion mass tolerances were 10 ppm for all MS acquired and fragment ion mass tolerance was 0.02 Da for all MS2 spectra. The search results were automatically processed at the FDR 1% at both the protein and peptide. The unique peptide included in the protein group was used for quantification. Proteins with all peptides SILAC ratios greater than or equal to 2 were considered as candidate interacting proteins.

### Surface plasmon resonance

Equilibrium dissociation constant ( *K*_D_) for sitagliptin-binding target proteins were determined by surface plasmon resonance (SPR) on a Biacore 8K instrument (Cytiva). Proteins were immobilized to theoretical resonance units (RU) on CM5 sensor chips (Cytiva). The binding kinetics was performed with a flow rate of 30 μL/min at 25°C. Biotin-PEG5-sitagliptin and biotin-PEG5 were dissolved in ddH _2_O and serially diluted (3.9–500 μM) in PBS buffer supplemented with 0.01% Tween 20 and injected over the sensor surface. All data were subtracted from blank injections and analyzed using Biacore 8K evaluation software (Cytiva) fitting to the 1:1 kinetic binding model. The kinetic binding curves for each candidate target proteins were generated using Prism 9 (GraphPad, San Diego, USA).

### Computational system arrangement

On the initial crystal structure of LYPLAL1 (PDB IDs: 3U0V, resolution: 1.72 Å), the substrates sitagliptin and 4-nitrophenyl acetate (PNPA) were docked using the software *AutoDock Vina*. AutoDock [21]. Similarly, sitagliptin and ATP were docked on the initial crystal structure of TCP1 (PDB: 7LUM, resolution: 4.50 Å). The substrate configuration was chosen based on the lowest energy provided by the software. Proteins were protonated using the *H++ web server* [22]. The pH was set at 7.5 to match the experimental conditions. The system preparation, minimization, molecular dynamic (MD) simulation, and the Molecular Mechanics Poisson-Boltzmann Surface Area (MMPBSA) were performed using AMBER-18 package. [23]. The enzyme-substrate coordinate and topology files were generated by the *tleap* module. The system was solvated using TIP3P water filled in a cubic box with the setting of an external water layer thickness of 10 Å from the protein surface. Chloride ions were added to neutralize the system.

### Minimization and molecular dynamic (MD) simulation

All simulations were conducted using the parallel version of *PMEMD*. *cuda*. Initially, the solvated system was relaxed by energy minimization. Using the Langevin thermostat, the system temperature was increased from 0 to 300 K for 100 ps, while the collision frequency was set to 2 ps ^–1^. To adjust the density under constant pressure and temperature (NPT), a 50-ps equilibration step was conducted. Prior to the MD simulation, a 5-ns equilibration step was completed. The MD simulations were conducted using ff14SB and GAFF force fields and the snapshots were extracted every 1000 steps in 2-fs integration time gaps. No restraints were applied during the 80-ns simulations.

### Free energy determined by MMPBSA

The binding free energy was calculated based on 100 snapshots (on LYPLAL1) and 50 snapshots (on TCP1) from 80 ns trajectories using MMPBSA approach [24]. The root-mean-square deviation (RMSD) and distance analysis was conducted using the CPPTRAJ toolset from AmberTools18 [25].

### Mutation analysis of binding sites

The predicted key-binding residue within the sitagliptin binding domain of TCP1 was mutated to alanine. Kinetic analysis was performed by SPR on mutant TCP1, while wild-type TCP1 was used as a control.

### Protein-protein interaction network construction

The Search Tool for the Retrieval of Interacting Genes/Proteins (STRING) website ( https://string-db.org/) was used to generate a protein-protein interaction network of CCAR2. After CCAR2 was imported into STRING, a protein-protein interaction (PPI) network was generated. Medium confidence was set at 0.400 as the minimum required interaction score.

### Chemistry

All reagents were commercially available and used without further purification. ^1^H nuclear magnetic resonance (NMR) spectra were recorded on an Agilent-400 instrument (Agilent, Santa Clara, USA) at 400 MHz. ^13^C NMR spectra were recorded on a Bruker AM-400 instrument (Bruker, Billerica, USA) at 101 MHz. Chemical shifts ( *δ*) were expressed in parts per million (ppm) relative to residual solvent as an internal reference for ^1^H and ^13^C NMR. Coupling constants ( *J*) were reported in hertz unit (Hz) and coupling patterns were described as singlet (s), doublet (d) and triplet (t). High resolution mass spectra (HRMS) were carried out on an Agilent Technologies 6230 LC-MS with (ESI-TOF) mode. Melting points (m.p.) were determined on a Büchi M-565 melting point apparatus (Büchi, New Castle, USA). HPLC analysis of all final biological testing compounds was performed on a Waters ACQUITY UPLC H-Class system with a Waters ACQUITY QDa system (Waters, Milford, USA) operating in the electrospray ionization (ESI) mode with a flow rate of 0.6 mL/min and a gradient of eluting with H _2_O (with 0.1% trifluoroacetic acid) and CH _3_CN. The method used is as follows: 7000 psi, flow rate =0.6 mL/min, eluent: t =0 min, 95% H _2_O; t =0.5 min, 95% H _2_O; t =3.5 min, 5% H _2_O; t =4.5 min, 5% H _2_O; t =5.0 min, 95% H _2_O, total acquisition time =5.0 min. Purity of all final testing compounds was based on the integrated UV chromatogram at 220 nm. The synthesis of biotin-PEG5-sitagliptin is shown in Supplementary Figure S1.

## Results

### Identification of sitagliptin binding proteins by AP-MS

**Figure 1:**
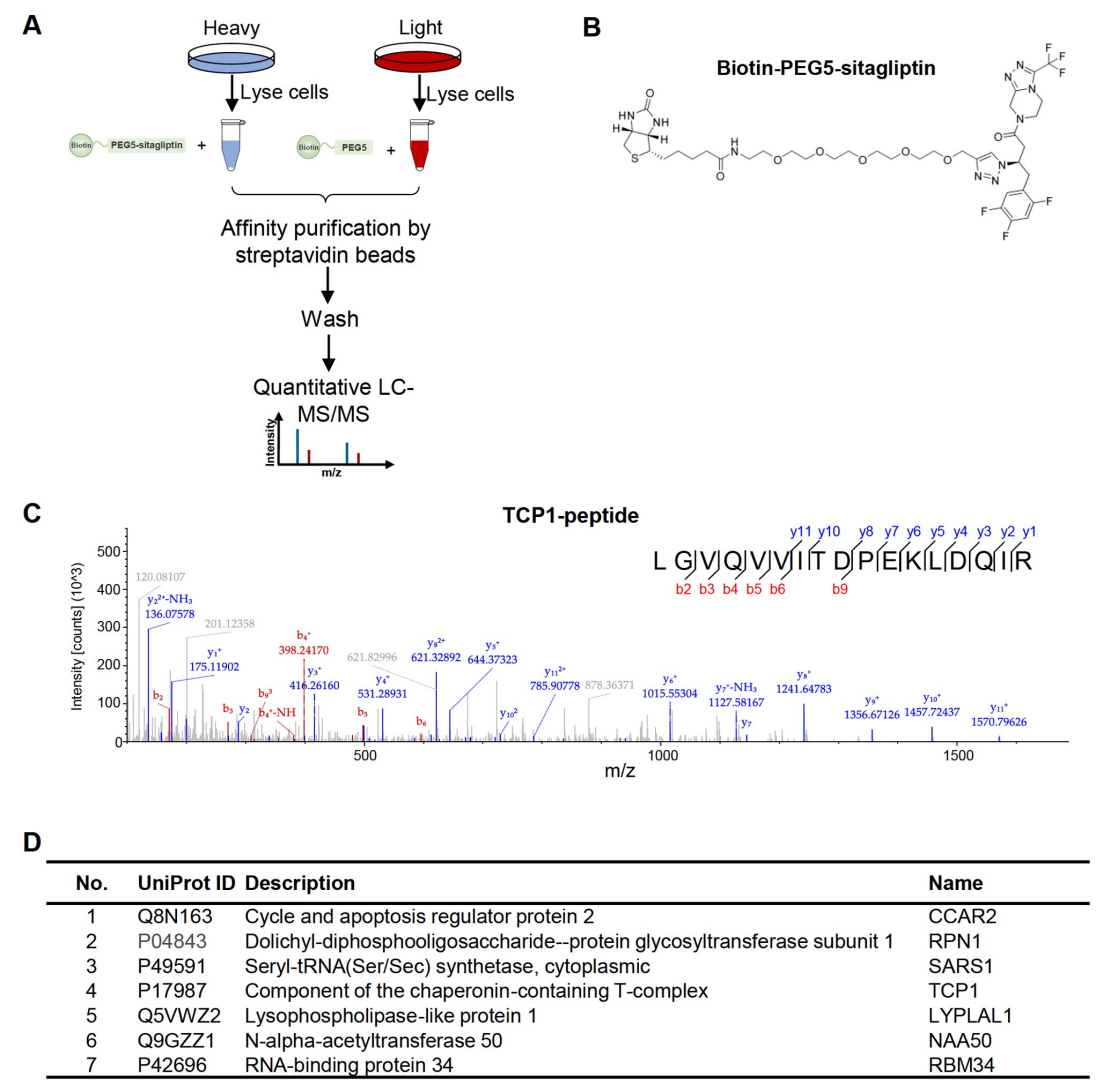
Figure 1 Identification of sitagliptin binding proteins by AP-MS (A) AP-MS workflow for the identification of sitagliptin binding proteins. (B) The structure of biotin-PEG5-Sitagliptin. (C) A peptide of TCP1 identified by LC-MS/MS. (D) High-confidence interacting proteins (proteins were obtained with heavy/light intensity ratio>2 in SILAC experiments).

To identify sitagliptin binding proteins, we applied SILAC coupled with AP-MS to enrich the binding proteins ( Figure 1A). Total HEK293T cell lysate was prepared under non-denaturing condition. Biotin-PEG5-sitagliptin ( Figure 1B) was incubated with heavy stable isotope labeled cell lysate, and biotin-PEG5 was incubated with light stable isotope labeled cell lysate as a control. After incubation, binding proteins were enriched by streptavidin agarose beads and identified by LC-MS/MS analysis. A total of seven high-confidence binding proteins compared with that of the control group (proteins were obtained with heavy/light intensity ratio>2 in SILAC experiments) were identified, namely, CCAR2, RPN1, SARS1, TCP1, LYPLAL1, NAA50, and RBM34 ( Figure 1D). As an example, the TCP1 peptide detected by LC-MS/MS was shown ( Figure 1C). Detected peptides of the other six proteins were shown in Supplementary Figure S2.

### Validation of sitagliptin binding proteins by SPR

**Figure 2:**
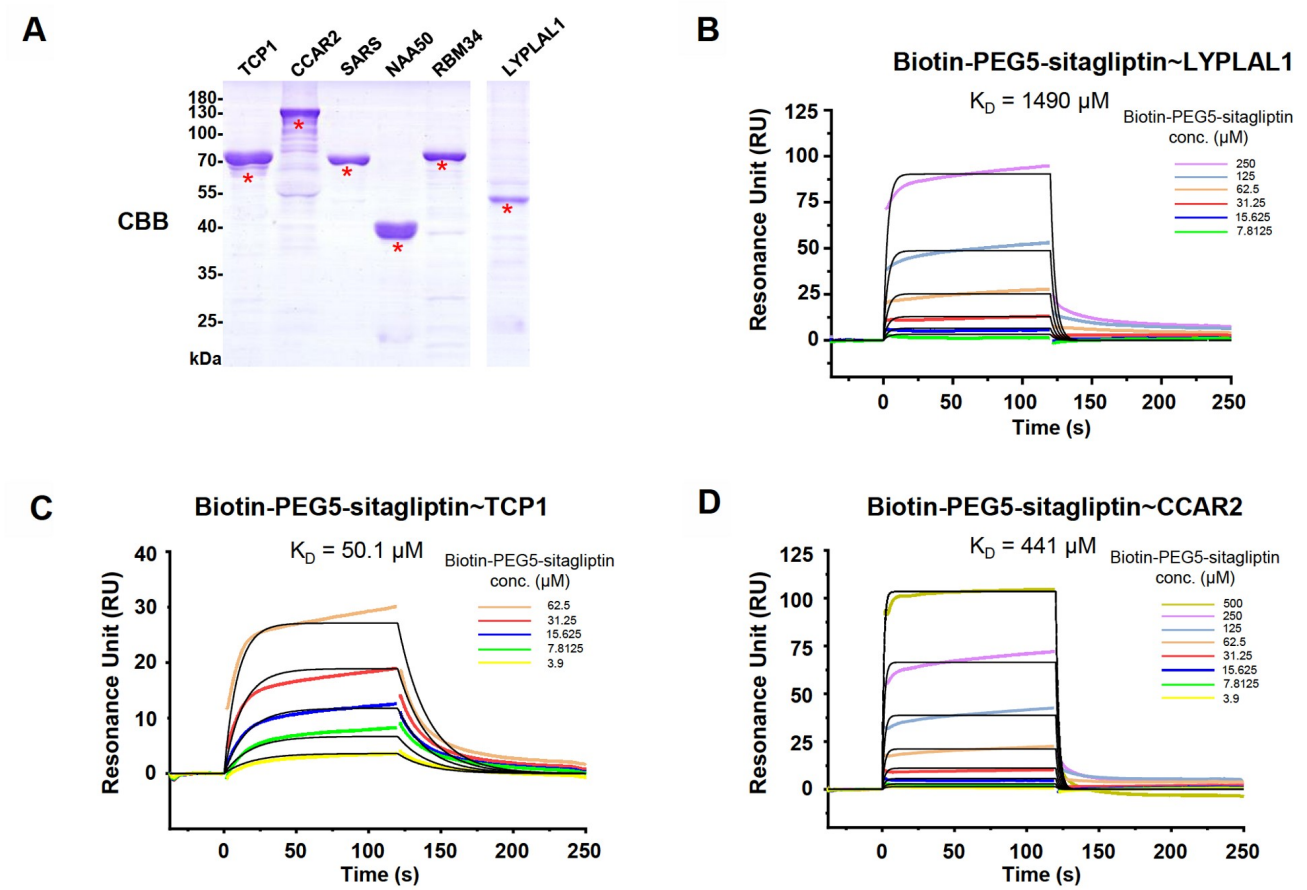
Figure 2 Validation of interactions between sitagliptin and its binding proteins by SPR analysis (A) Preparation of six proteins for SPR analysis. (B‒D) Binding affinities between sitagliptin and three interacting proteins, i. e., (B) LYPLAL1, (C) TCP1, and (D) CCAR2.

To validate sitagliptin binding proteins, we successfully purified six out of seven proteins (TCP1, CCAR2, SARS, NAA50, RBM34 and LYPLAL1) and examined their interactions with sitagliptin by surface plasmon resonance (SPR) ( Figure 2A). We measured the binding and dissociation of biotin-PEG5-sitagliptin with purified proteins immobilized on the research grade CM5 chip. The response signal for each protein was obtained at gradient concentrations of biotin-PEG5-sitaglpitin (3.9‒500 μM). PBS was used throughout the experiment as baseline reference to determine systematic drifts. In order to rule out the possible non-specific binding caused by the linker (biotin-PEG5), we also measured the binding affinity of the linker to each protein under the same condition as negative controls ( Supplementary Figure S3A‒C). Our results revealed that 3 proteins, namely lysophospholipase-like 1 (LYPLAL1), T-complex protein 1 subunit alpha (TCP1), and cell cycle and apoptosis regulator protein 2 (CCAR2; also known as deleted in breast cancer 1, DBC1), indeed bound to sitagliptin ( Figure 2B‒D).

Both LYPLAL1 ( Figure 2B) and CCAR2 ( Figure 2D) underwent the fast-binding and fast dissociation reaction with the ligand. LYPLAL1 is one of three human cytosolic acyl protein thioesterases [26]. LYPLAL1-ligand complex had an equilibrium constant ( *K*_D_) value of 1440 μM, with associated rate constant ( *K*_a_) value and dissociation rate constant value ( *K*_d_) being 195 M ^–1^S ^–1^ and 0.396 S ^–1^, respectively. The *K*_a_, *K*_d_, and *K*_D_ of CCAR2-ligand complex were 821 M ^‒1^S ^‒1^, 0.513 S ^‒1^, and 441 μM, respectively. CCAR2 is a core component of the DBIRD complex and participates in diverse biological processes including heterochromatin formation, transcription, mRNA splicing, apoptosis, and cell proliferation [27]. Compared with LYPLAL1- and CCAR2-ligand complex, the K _a_ of sitagliptin (977 M ^‒1^S ^‒1^) with TCP1 was approximately 5 folds of that with LYPLAL1 (195 M ^‒1^S ^‒1^) and comparable to that with CCAR2 (821 M ^‒1^S ^‒1^); however, the *K*_d_ of sitagliptin with TCP1 (0.049 S ^‒1^) was about 8-fold less than that with LYPLAL1 (0.396 S ^‒1^) and 10-fold less than that with CCAR2 (0.513 S ^‒1^), resulting in a 40- to 10-fold higher target affinity with TCP1 ( *K*_D_ =50.1 μM) compared with the other two binding proteins ( Figure 2C). TCP1 is a subunit of TCP1-containing ring complex (TRiC), which is a large (1 MDa), ring-shaped chaperonin belongs to Group II chaperonins and exists in the cytosol in eukaryotic cells [28].

### Molecular docking and MD simulations

In the study, the crystal structures of LYPLAL1 (PDB IDs: 3U0V, resolution: 1.72 Å) and TCP1 (PDB: 7LUM, resolution: 4.50 Å) were used to predict the mode of sitagliptin binding. Replicates of 20 ns MD simulation was conducted on the protein complex. Based on the RMSD analysis of the C-alpha carbon backbone, the most stable trajectory was selected for 80 ns simulation. MMPBSA analysis was conducted to measure the substrate binding. We found that sitagliptin could bind to LYPLAL1 and TCP1 stably in their active sites.

**Figure 3:**
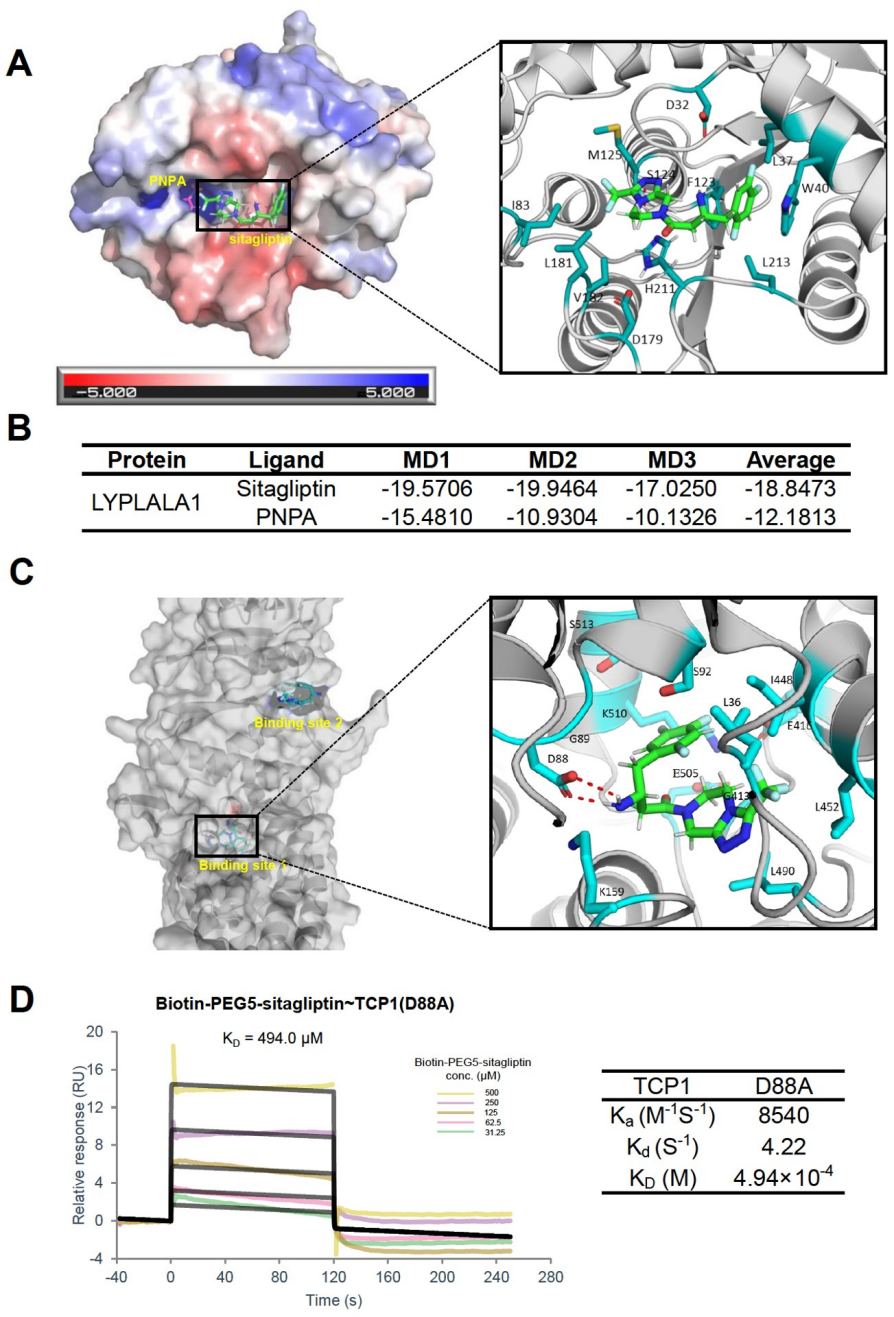
Figure 3 Molecular docking results of sitagliptin on LYPLAL1 and TCP1 (A) 4-nitrophenyl acetate (PNPA) and sitagliptin docked on the active site of LYPLAL1. Red and blue in the protein surface indicate negative and positive electrostatic potentials, respectively. The interactions of sitagliptin with the key residues in the active cavity of LYPLAL1 are highlighted. (B) Binding enery of sitagliptin and PNPA on LYPLAL1 (PDB ID: 3U0V) (scores in kcal/mol) of three times repeated MD simulations. (C) Molecular docking results of sitagliptin on TCP1. Two possible binding sites are shown, and key residues of sitagliptin binding to TCP1 in site 1 are highlighted. (D) Validation of the interaction between sitagliptin and TCP1 (D88A) by SPR analysis.

LYPLAL1 has a typical α/β hydrolase fold and a classical catalytic triad formed by three key residues: Ser124, Asp179 and His211 [29]. Distinct positive charge surrounding the catalytic serine indicated that a small molecule with at least partial negative charge was most likely to bind to LYPLAL1 ( Figure 3A). Furthermore, LYPLAL1 was reported to contain an open, solvent-accessible core with a shallow hydrophobic tunnel, which allowed physical entrapment of substrate once entered [26]. To determine if sitagliptin can stably bind to LYPLAL1 in the active site, the known substrate of LYPLAL1, *i.e*., 4-nitrophenyl acetate (PNPA), was selected to be docked to LYPLAL1 as a reference of a comparison study throughout the MD simulation [26]. The MD simulations of both LYPLAL1-sitagliptin and LYPLAL1-PNPA complexes were carried out for 20-ns MD simulation, and both complexes reached their equilibrium within 20 ns. Additionally, three repeated 80-ns MD simulations were performed on each complex. Structural and dynamical properties of sitagliptin and PNPA were analyzed throughout all MD simulations. RMSD values were calculated to evaluate the deviation of the structure throughout simulations from the starting structure, and the results indicated that both complexes were stable during the entire course of the simulations ( Supplementary Figure S4A). Figure 3A highlighted representative structures of LYPLAL1-sitagliptin and LYPLAL1-PNPA.

Along with the docking scores, the average MMPBSA decomposition energies of both complexes equally distributed throughout the three repeats of 80-ns MD simulations were also monitored. Figure 3B shows that the average binding energy of LYPLAL1-sitagliptin (‒18.8473 Kcal/mol) was higher than that of LYPLAL1-PNPA (‒12.1813 Kcal/mol). The cavity is naturally spacious and hydrophilic to accommodate the solvent, as the hydrolase requires water to complete the reaction. Therefore, throughout the simulation, PNPA was often found to be surrounded by solvent, while lacking a strong form of residue-substrate interactions. Sitagliptin is much longer than PNPA. It binds to the catalytic residues and key residues of the exterior of the cavity (Asp32, Try40, Phe123, His211, Leu181, Ser124, Leu37, Met125, Asp179, Ile83, Val182 and Leu213). Particularly, π-π stacking interaction was present on both sides of the terminal phenyl moiety of sitagliptin with Trp40 and Phe123, which stabilized the sitagliptin binding. A strong hydrogen bond was formed between the triazole ring of sitagliptin and the residue Ser124 (shown in Figure 3A). These results suggest that sitagliptin can bind to the active site throughout a large surface in a stable orientation, and therefore may disturb the catalytic activity of LYPLAL1.

Next, we asked how sitagliptin binds to TCP1, one of subunits of TRiC complex. TRiC consists of two identical stacked rings, with eight homologous subunits in each ring. Each subunit contains three domains: an equatorial ATP-binding domain, an apical substrate-binding domain, and a hinge-like intermediate domain [30]. TRiC folds proteins in an ATP-dependent way. Specifically, a built-in lid protruding from the apical domain of each subunit can open or close upon an elaborate conformational cycle triggered by ATP binding or hydrolysis. During this process, the substrate is encapsulated within the central chamber of TRiC and then folds. To predict if sitagliptin affects the binding of ATP to TCP1, we used ATP as a reference molecule and performed molecular docking as described above. Two possible binding sites for sitagliptin were selected and shown in Figure 3C. Binding site 1 of sitagliptin is located in the equatorial domain, sharing the same site with ATP. Binding site 2 is in the intermediate domain. Based on the MMPBSA analysis, sitagliptin was bound to binding site 1 and 2 at –21.9092 and –19.8047 kcal/mol, respectively. ( Table 1). These results indicated that both sites could be probable for the stable binding of sitagliptin ( Supplementary Figure S4B). Here we highlighted the binding site 1 ( Figure 3C), which showed that sitagliptin is stabilized by hydrogen bonds and electrostatic interactions with key residues Leu36, Asp88, Gly89, Ser92, Lys159, Gly413, Glu416, Ile448, Leu452, Leu490, Glu505, Lys510, and Ser513 ( Figure 3C). Hydrogen bond pairs are formed between the linker amide of sitagliptin and the universally conserved catalytic Asp88, located in the conserved phosphate-binding loop (P-loop) GDGTT-motif of the ATP-binding pocket. This mode of binding of sitagliptin to TCP1 suggests a possible interference of ATP binding. To validate the binding mode of sitagliptin-TCP1, we chose the key residue Asp88 and performed site-directed mutagenesis experiments followed by SPR analysis. The mutagenesis study demonstrated that TCP1 D88A mutant resulted in a nearly 10-fold decrease of sitagliptin binding affinity ( *K*_D_ =494.0 μM) ( Figure 3D) compared with that of the wild-type TCP1 ( *K*_D_ =50.1 μM). This result confirmed the role of Asp88 in sitagliptin-TCP1 binding.

**Table:** **Table 1** MMPBSA free energy of binding analysis of sitagliptin and ATP on TCP1 (PDB ID: 7LUM)

	^1^ΔG _vdw_(Kcal/mol)	^2^ΔG _ele_(Kcal/mol)	^3^ΔG _polar_(Kcal/mol)	^4^ΔG _nonpolar_(Kcal/mol)	^5^ΔG _gas_(Kcal/mol)	^6^ΔG _solv_(Kcal/mol)	^7^ΔG _total_(Kcal/mol)
Sitgliptin Binding site 1	‒41.4372±0.4860	‒23.7968±0.8193	47.3448±1.0197	‒4.0200±0.0182	‒65.2340±0.7700	43.3248±1.0147	‒21.9092±0.6966
Sitgliptin Binding site 2	‒35.0029±1.8155	‒14.4999±3.3527	33.1287±3.3590	‒3.4307±0.1101	‒49.5028±3.2175	29.6981±3.3112	‒19.8047±1.4747
ATP binding site	‒41.2176±0.7820	‒176.7184±5.1684	199.6400±4.6757	‒4.0574±0.0232	‒217.9360±5.0541	195.5826±4.6711	‒22.3534±1.0186

Energy components include: ^1^Van der Waals contribution. ^2^Electrostatic energy. ^3^Electrostatic contribution to the solvation free energy. ^4^Nonpolar contribution to the solvation free energy. ^5^Gas phase energy. ^6^Solvation energy. ^7^Total.

### Constructing protein interaction network of CCAR2

**Figure 4:**
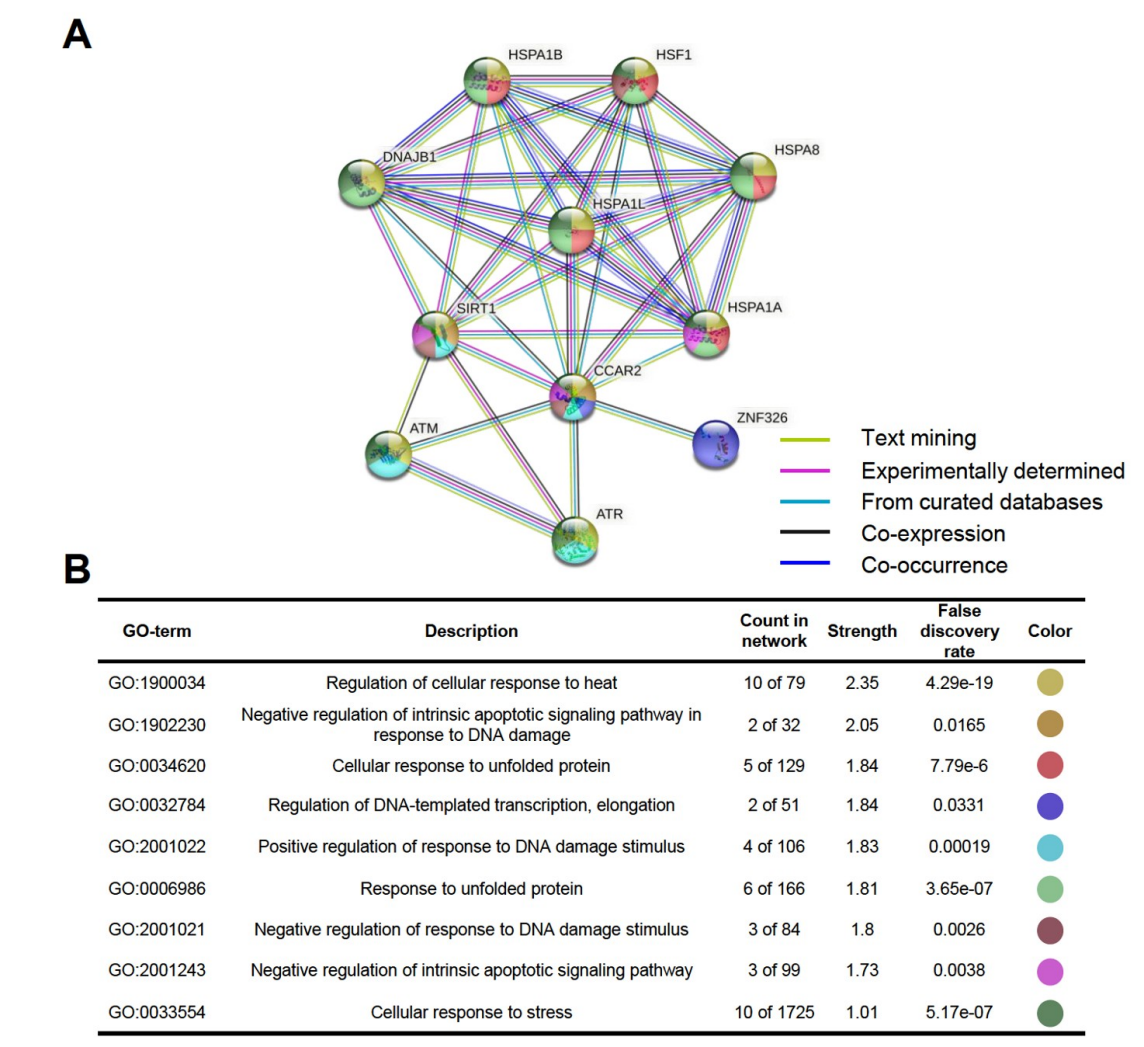
Figure 4 Protein-protein interaction network of CCAR2 Color-coded nodes using a split-pie chart indicating enriched GO terms.

Since no complete crystal structure of CCAR2 is available yet, we are unable to perform docking analysis. Instead, we evaluated the functional interaction between CCAR2 and other interactors by construing a PPI network using STRING ( Figure 4A). In this network, nodes represent interactors and edges indicate the type of interaction evidences which include interactors from text and curated databases mining, experimentally determined interactors, co-expression and co-occurrence. The top 10 interactors include HSPA1B, HSF1, DNAJB1, HSPA8, HSPA1L, SIRT1, HSPA1A, ATM, ZNF326, and ATR. The analyses highlighted the enriched biological process for CCAR2, and the most important enriched terms with false discovery rates (FDRs) lower than 0.05 were: regulation of cellular response to heat, negative regulation of intrinsic apoptotic signaling pathway in response to DNA damage, regulation of DNA-templated transcription and elongation, positive regulation of response to DNA damage stimulus, negative regulation of response to DNA damage stimulus, negative regulation of intrinsic apoptotic signaling pathway, and cellular response to stress ( Figure 4B).

## Discussion

Although sitagliptin shows excellent therapeutic outcome, it has adverse effects [16], including nasopharyngitis, upper respiratory infection, and headache. In addition, cases have been reported that sitagliptin is likely correlated with angio-oedema and worsening renal function [ 15, 16] . Therefore, identification of new targets can help to systemically evaluate the potential side effects of sitagliptin. Moreover, as it has been reported that sitagliptin may serve as a potential drug for the treatment of cardiovascular diseases [20], cancer [17], and even COVID-19 [31]. Undoubtedly, identifying candidate targets will help uncover more therapeutic functions of sitagliptin. In the present study, we identified seven high-confidence sitagliptin-interacting proteins by AP-MS. Three proteins, *i*. *e*., LYPLAL1, TCP1, and CCAR2, were confirmed to have weak interaction with sitagliptin. It is sufficient for the weak binder to propel a response if the local concentration is high enough to drive the equilibrium and leads to considerable bound ligand. As far as drug development is concerned, weak binders can bind to their targets in a more transient way with reduced adverse side reactions [ 32, 33] . Therefore, those weak-affinity molecules may serve as a new range of drug candidates [33].

Both DPP-4 and LYPLAL1 fall into the large α/β hydrolase (ABH) fold superfamily, which includes proteases, haloperoxidases, esterases, thioesterases, lipases, epoxide hydrolases, and dehalogenases, and they play catalytic roles in various metabolic reactions [34]. Although members of the ABH family share low sequence identity, they have highly conserved three-dimensional core architectures. The catalytic triad, which consists of a catalytic acid (Asp or Glu), a nucleophile (Ser, Asp or Asn), and His, is the most conserved feature of ABH [35]. X-ray crystallography studies showed that sitagliptin, together with other commercially available DPP-4 inhibitors such as vildagliptin, alogliptin, and saxagliptin, all bind essentially to the same catalytic site in DPP-4, occupying the S1 pocket and Glu 205, Glu206, and Arg125, thus behaving as competitive inhibitors and protect GLP-1 and GIP from rapid inactivation [36]. Our LYPLAL1-sitaglpitin complex docking result showed the stable hydrogen interaction formed between sitagliptin and the catalytic residue Ser124, indicating that sitagliptin may occupy the catalytic triad region of LYPLAL1 in a similar way, as it binds to the pocket of DPP-4. Therefore, it may serve as an inhibitor to decrease the basal activity of LYPLAL1, and overdose of sitagliptin may cause adverse effects in T2DM treatment. So far, knowledge of the function of LYPLAL1 remains limited. Although genome-wide identification has shown that genetic variants near LYPLAL1 is associated with insulin resistance, and individuals carrying relative alleles are at higher risk for T2DM [ 37, 38] , these variants falling outside the coding region of LYPLAL1 are unable to explain whether activation or inhibition of LYPLAL1 are linked to T2DM risk. Further experiments are required to determine the precise effects of sitagliptin on LYPLAL1 both *in vitro* and *in vivo*.

TCP1 is an important subunit of TRiC [39]. TRiC was originally assumed to regulate the folding of a narrow class of structural proteins, such as actin and tubulin [40]. Later on, the list of anticipated substrates in the eukaryotic cell has become longer. It has been suggested that about 10% of newly synthesized proteins interact with TRiC upon ATP-dependent activation [9]. Reissmann *et al*. [40] reported a significant asymmetry in the ATP-driving cycle where a gradient of affinities governs ATP hierarchically binding to TRiC subunits, and TCP1 is one of the four subunits (TCP1, CCT2, CCT4, and CCT5) which have high ATP affinities required for the viability of TRiC. In our study, SPR results showed micromolar binding affinity of sitagliptin towards TCP1. The structural simulation results suggested that sitagliptin could bind to the equatorial ATP-binding domain. Based on the MD simulation result, we speculate that sitagliptin might hinder ATP binding and hydrolysis, and may interfere the change in conformation and protein folding. TCP1 and the entire TRiC complex have been considered as potential therapeutic targets in breast cancer [41]. Previous studies have shown that TCP1 plays an essential role in determining the overall survival (OS) of patients with breast cancer. Guest *et al*. [41] identified that TCP1 together with CCT2 (TRiC subunit) are commonly altered in breast cancer and essential for the proliferation of breast cancer cells. In addition, there is a strong link between some known substrates of TRiC and cancer. For example, PLK1, a substrate of TRiC, is overexpressed in breast cancer and is considered as a diagnostic prediction model [42]. The substrate tubulin is also thought to have an inhibitory effect on the anticancer drug paclitaxel [43]. These studies all suggest that TRiC inhibitors may have a positive effect on the treatment of breast cancer and other cancers.

Our *in vitro* investigation also showed that sitagliptin binds to CCAR2 with weak affinity. It has been observed that expression of CCAR2 is decreased in T2DM patients’ peripheral blood mononuclear cells [44]. CCAR2 was first described to bind to the histone deacetylase (HDAC), SIRT1, and used as a negative regulator of SIRT1’s deacetylase activity [45]. As SIRT1 is known to regulate glucose/lipid homeostasis and energy expenditure in the liver, CCAR2, the endogenous inhibitor of SIRT1, may have potential implications in T2DM pathogenesis [44]. Indeed, CCAR2 participates in multiple metabolic pathways that related to the regulation of glucose homeostasis. Aberrant upregulation of liver gluconeogenesis can lead to glucose intolerance, which is one of the hallmarks of T2DM [46]. Physiologically, *CCAR2*-knockout mice displayed higher gluconeogenesis [46], and a substantial number of genes involved in glucose homeostasis are co-regulated by CCAR2 [47]. Further experiments are needed to demonstrate whether sitagliptin has an auxiliary function in maintaining glucose homeostasis by binding to CCAR2 to regulate gluconeogenesis. With regards to cancer, CCAR2 is also a research hotspot, as it plays a pleiotropic role as both tumor suppressor and tumor promoter [ 27, 48, 49] . Increasing evidence has shown that CCAR2 upregulation is associated with poor survival among tumor patients with breast carcinoma, gastric carcinoma, esophageal cancer, etc. [48], and CCAR2 may serve as a tumor diagnostic or therapeutic target [27]. A number of proteins have been confirmed to interact with CCAR2, however, not much is known about the function and the molecular cues modulating the interactions between CCAR2 and its binding proteins. The mechanism needs to be further investigated to find out whether and how sitagliptin manipulates CCAR2, especially the involvement of CCAR2 in regulating glucose homeostasis and tumorigenic growth.

In summary, in the present study we globally profiled the interacting proteins of sitagliptin. We validated that sitagliptin binds to three cellular proteins (LYPLAL1, TCP1, and CCAR2) with micromolar affinities, and these proteins are closely related to glucose homeostasis, proteins folding, and tumorigenic growth. Our findings provide insights into the sitagliptin-targets interplay and indicate the potential risk of overdose sitagliptin in the treatment of T2DM, as well as the pleiotropic therapy effects of sitagliptin. Further studies, especially functional studies, are needed to confirm the interactions *in vivo*, and to explore the possible application of sitagliptin in the treatment of other diseases.

## Supplementary Data

Supplementary data is available at *Acta Biochimica et Biophysica Sinica* online.

## Supporting information

22079supplementary_figures

22079Table_1
