# Supplementary material for: Identification of sitagliptin binding proteins by affinity purification mass spectrometry: Sitagliptin binding proteins identified by affinity purification mass spectrometry
Source: Acta Biochim Biophys Sin (Shanghai). 2022 Oct 9;54(10):1453–63. doi: 10.3724/abbs.2022142 (PMC9827809; doi:10.3724/abbs.2022142)
Supplement: 22079supplementary_figures [file 22079supplementary_figures.pdf]

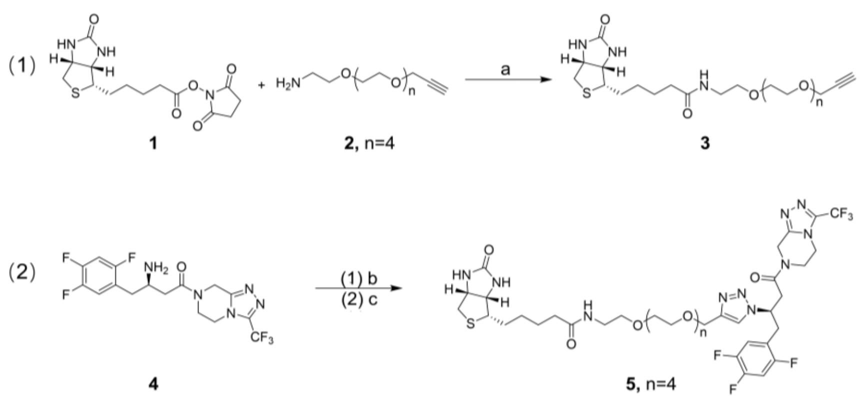

Supplementary Figure S1. The synthesis of biotin-PEG5-sitagliptin Reagent and conditions: (a) TEA, DMF, room temperature; (b) FSO_2N_3 (0.3M in MTBE), $KHCO_3$ (2M in H_2O), DMF, room temperature; (c) compound **3**, sodium ascorbate, $CuSO_4 \cdot 5H_2O$, DMF, H_2O, 50ºC. The detail methods are shown as the follows:

N-(3,6,9,12,15-Pentaoxaoctadec-17-yn-1-yl)-5-((3aS,4S,6aR)-2-oxohexahydro-1H-thieno[3,4-d]imidazol-4-yl)pentanamide (3)

To a solution of biotin-NHS (**1**) (912 mg, 2.67 mmol) in DMF (10 mL) was added compound **2** (500 mg, 2.67 mmol) and TEA (689 μL, 5.34 mmol). The reaction mixture was stirred at room temperature overnight. The solvent was then removed *in vacuum* and the residue was purified by silica gel column chromatography (dichloromethane/methanol=10/1) to afford compound **3** (822 mg, 90%) as a light yellow solid. ^{1}H NMR (400 MHz, DMSO-d_6): δ 7.84 (t, J=5.6 Hz, 1H), 6.43 (s, 1H), 6.36 (s, 1H), 4.32-4.28 (m, 1H), 4.14-4.10 (m, 3H), 3.55-3.51 (m, 16H), 3.43 (t, J=2.4 Hz, 1H), 3.37 (t, J=6.0 Hz, 2H), 3.20-3.15 (m, 2H), 3.11-3.07 (m, 1H), 2.84-2.79 (m, 1H), 2.59-2.55 (m, 1H), 2.06 (t, J=7.2 Hz, 2H), 1.65-1.23 (m, 6H) ppm; ^{13}C NMR (101 MHz, DMSO-d_6): δ 172.0, 162.6, 80.3, 77.0, 69.7, 69.6, 69.5, 69.4, 69.1, 68.4, 60.9, 59.1, 57.4, 55.3, 39.8, 38.4, 35.0, 28.1, 27.9, 25.2 ppm.

N-(1-(1-((R)-4-Oxo-4-(3-(trifluoromethyl)-5,6-dihydro-[1,2,4]triazolo[4,3-a]pyrazin-7(8H)-yl)-1-(2,4,5-trifluorophenyl)butan-2-yl)-1H-1,2,3-triazol-4-yl)-2,5,8,11,14-pentaoxahexadecan-16-yl)-5-((3aS,4S,6aR)-2-oxohexahydro-1H-thieno[3,4-d]imidazol-4-yl)pentanamide (5)

To a solution of compound **4** (0.2 mmol) in DMF (2 mL) was added $KHCO_3$ (2 M in H_2O, 0.4 mL, 0.8 mmol) aqueous and FSO_2N_3 (0.3 M in MTBE, 0.8 mL, 0.24 mmol). After stirred at room temperature for 2 h, to the mixture was added sodium ascorbate (158 mg, 0.8 mmol), biotin-PEG5-alkyne **3** (82.6 mg, 0.2 mmol) and $CuSO_4 \cdot 5H_2O$ (10 mg, 0.04 mmol). The mixture was then heated to 50ºC. After stirred for 2 h, the

mixture was cooled to room temperature and evaporated *in vacuum*. The residue was purified by flash column chromatography (0% acetonitrile, 100% H_2O to 100% acetonitrile, 40 min) to afford compound **5** (118 mg, 63%) as a light yellow viscous solid. ^{1}H NMR (400 MHz, DMSO-d_6): δ 8.04 (d, *J*=10.4 Hz, 1H), 7.84 (s, 1H), 7.49-7.35 (m, 1H), 7.26-7.09 (m, 1H), 6.43 (s, 1H), 6.36 (s, 1H), 5.29-5.17 (m, 1H), 5.06-4.74 (m, 2H), 4.44 (s, 2H), 4.30-3.83 (m, 6H), 3.63-3.38 (m, 18H), 3.29-3.08 (m, 7H), 2.83-2.80 (m, 1H), 2.58-2.56 (m, 1H), 2.06 (s, 2H), 1.63-1.28 (m, 6H) ppm; ^{13}C NMR (101 MHz, DMSO-d_6): δ 172.0, 168.2 (d, *J*=5 Hz), 162.6, 155.8 (dd, *J*=244, 10 Hz), 150.7 (d, *J*=11 Hz), 148.0 (dt, *J*=249, 13 Hz), 144.4 (dd, *J*=13, 3 Hz), 143.3 (d, *J*=4 Hz), 142.3 (q, *J*=18 Hz), 123.5 (d, *J*=24 Hz), 119.8 (ddt, *J*=195, 19, 4 Hz), 118.4 (q, *J*=271 Hz), 105.6 (dd, *J*=29, 21 Hz), 69.7, 69.6, 69.5, 69.5, 69.1, 68.5, 63.3, 61.0, 59.1, 57.2 (d, *J*=6 Hz), 55.3, 43.4, 42.9, 41.6, 40.8, 39.8, 38.4, 37.4, 36.9, 36.8, 35.0, 33.6, 28.1, 27.9, 25.2 ppm. HRMS (ESI): $[M+H]^+$ $C_{39}H_{53}F_6N_{10}O_8S$ calcd 935.3673, found 935.3666; HPLC: purity 97.2%, retention time 2.17 min

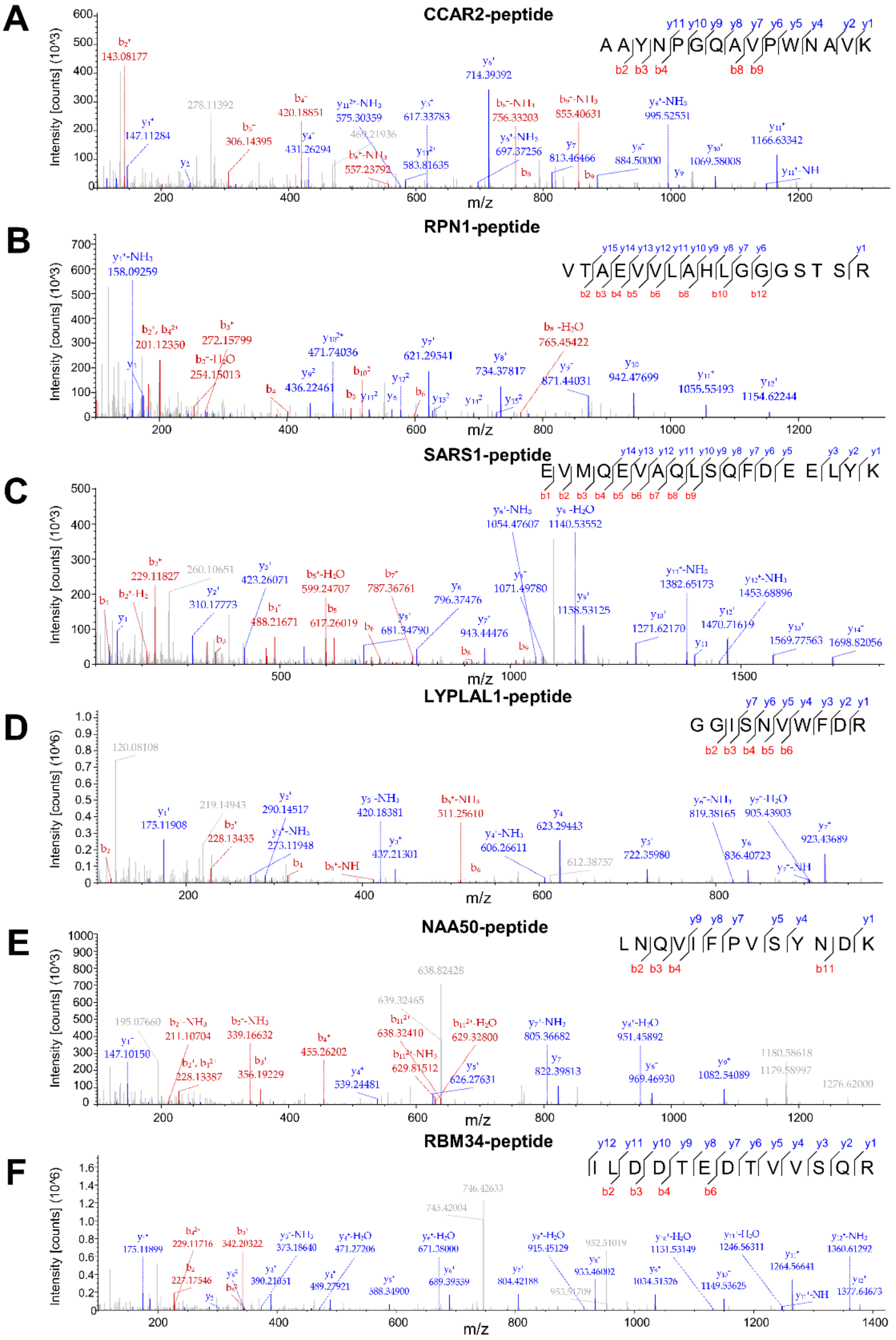

Supplementary Figure S2. Peptides of CCAR2 (A), RPN1 (B), SARS1 (C), LYPLAL1 (D), NAA50 (E), and RBM34 (F) identified by LC–MS/MS

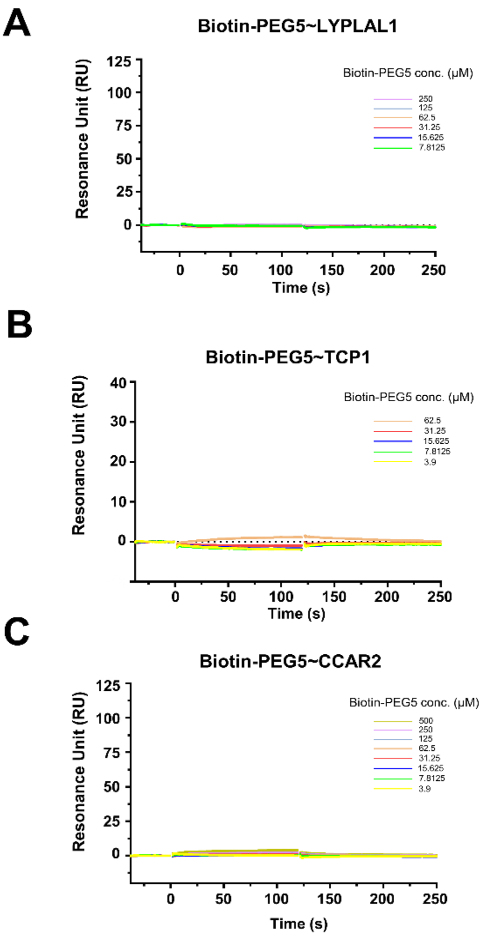

Supplementary Figure S3. Validation of interactions between biotin-PEG5 and identified interactors by SPR analysis as negative controls Identified interactors include (A) LYPLAL1, (B) TCP1, and (C) CCAR2.

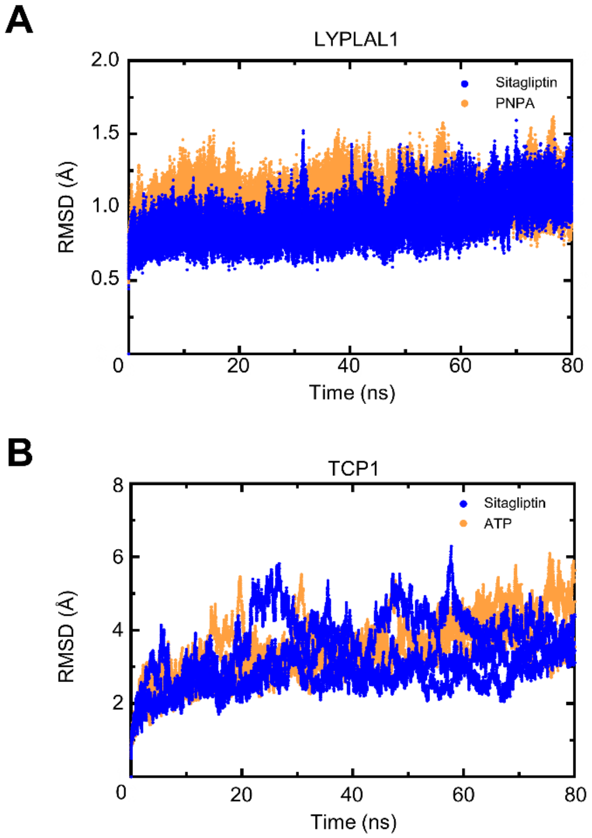

Supplementary Figure S4. RMSD values of receptors in the complexes along the 80-ns MD simulations (A) RMSD value of LYPLAL1 binding to sitagliptin (blue) and PNPA (yellow), respectively. (B) RMSD value of TCP1 binding to sitagliptin (blue) and ATP (yellow), respectively. Average values are from multiple MD simulations.
