# Supplementary material for: Identification of sitagliptin binding proteins by affinity purification mass spectrometry: Sitagliptin binding proteins identified by affinity purification mass spectrometry
Source: Acta Biochim Biophys Sin (Shanghai). 2022 Oct 9;54(10):1453–63. doi: 10.3724/abbs.2022142 (PMC9827809; doi:10.3724/abbs.2022142)
Supplement: 22079Table_1 [file 22079Table_1.pdf]

Table 1. MMPBSA free energy of binding analysis of sitagliptin and ATP on TCP1 (PDB ID: 7LUM)

	[1]ΔG_{vdw} (Kcal/mol)	[2]ΔG_{ele} (Kcal/mol)	[3]ΔG_{polar} (Kcal/mol)	[4]$\Delta G_{nonpolar}$ (Kcal/mol)	[5]ΔG_{gas} (Kcal/mol)	[6]ΔG_{solv} (Kcal/mol)	[7]ΔG_{total} (Kcal/mol)
Sitgliptin	−41.4372	−23.7968	47.3448	−4.0200	−65.2340	43.3248	−21.9092
Binding site 1	±0.4860	±0.8193	±1.0197	±0.0182	±0.7700	±1.0147	±0.6966
Sitgliptin	−35.0029	−14.4999	33.1287	−3.4307	−49.5028	29.6981	−19.8047
Binding site 2	±1.8155	±3.3527	±3.3590	±0.1101	±3.2175	±3.3112	±1.4747
ATP binding site	−41.2176	−176.7184	199.6400	−4.0574	−217.9360	195.5826	−22.3534
	±0.7820	±5.1684	±4.6757	±0.0232	±5.0541	±4.6711	±1.0186

Energy components include: [1]Van der Waals contribution. [2]Electrostatic energy. [3]Electrostatic contribution to the solvation free energy. [4]Nonpolar contribution to the solvation free energy. [5]Gas phase energy. [6]Solvation energy. [7]Total.
